# Knowledge and acceptance of COVID-19 vaccine among healthcare workers in Enugu metropolis, Enugu state, Nigeria

**DOI:** 10.3389/fpubh.2023.1084854

**Published:** 2023-06-22

**Authors:** Kelechi U. Imediegwu, Jude C. Abor, Chiamaka Q. Onyebuchukwu, Hilary I. Ugwu, Ogechi I. Ugwu, Udo Ego Anyaehie, Oluchi A. Onyia

**Affiliations:** ^1^Department of Orthopaedic Surgery, National Orthopaedic Hospital, Enugu, Nigeria; ^2^Final Year Medical Student, University of Nigeria (UNN), Nsukka, Enugu, Nigeria

**Keywords:** COVID-19 vaccine, knowledge, acceptance, hesitancy, healthcare workers, Enugu metropolis

## Abstract

**Background:**

COVID-19 disease spread at an alarming rate, and was declared a pandemic within 5 months from the first reported case. As vaccines have become available, there was a global effort to attain about 75% herd immunity through vaccination. There is a need to address the issue of vaccine hesitancy to COVID-19 vaccines especially in places such as Sub-Saharan African countries which have a high rate of background vaccine hesitancy.

**Objective:**

To determine the knowledge and acceptance of COVID-19 vaccines among healthcare workers (HCWs) in Enugu metropolis.

**Methods:**

A cross-sectional descriptive study of 103 HCWs in Enugu metropolis was done. Data was collected using structured online Google forms. Descriptive and inferential statistics was done using SPSS, and results were summarized into percentages and associations.

**Results:**

An acceptance rate of 56.2% was obtained among HCWs in Enugu metropolis. Positive predicators of acceptance include older age (*p* = 0.004, *X*^2^ = 13.161), marriage (*p* = 0.001, *X*^2^ = 13.996), and higher average level of income (*p* = 0.013, *X*^2^ = 10.766) as significant correlations were found. No significant association was found between educational level, religion, denomination nor occupation, and acceptance of vaccine. The major factor responsible for refusal was fear of side-effects.

**Discussion:**

The acceptance rate of COVID-19 vaccines among HCWs is still less than optimal. This population represents the most enlightened population on health related matters, hence if acceptance rate remains merely average that in the general population is expected to be worse. There is a need to address the fear of vaccine side-effects by inculcating more open and interactive methods of information dissemination, while also addressing the misconceptions or myths surrounding COVID-19 vaccines.

## Introduction

Like the other pathogens Severe acute respiratory syndrome coronavirus-1 (SARS-CoV-1) and Middle East respiratory syndrome coronavirus (MERS-CoV) that belong to the same Coronavirus family, the emergence of the novel *coronaviridae* shook the world like a storm (1). In the January of 2020, the World Health Organization (WHO) following the recommendations of the investigative team declared the disease to be a public health emergency of international concern and shortly afterwards a pandemic (2). The WHO also in conjunction with the different national health agencies in the member nations rallied to mount the highest possible medical defence. Africa was predicted to be the graveyard of the pandemic due to densely populated cities and the almost non-existent healthcare infrastructure but against all odds, the continent has almost escaped the grave effects of the disease seen in a couple of other places (3). The region has so far remained the least affected WHO region with about 9.3 million confirmed cases and 226,960 deaths as at December 2021 (4, 5). Globally as at December 2021, a total of 273,869,899 confirmed cases had been recorded with 5,352,069 deaths hence the case fatality rate was about 2%.

A major difficulty evident from the very beginning of the pandemic was the lack of efficacious therapeutics against the SARS-CoV-2 virus. Treatment was and still remains largely supportive and experimental (6). As a result of this, a drive to find an alternative solution led to the race to develop COVID-19 vaccines in order to achieve a global herd immunity. The first batch of vaccines were approved for use around December 2020 in the United Kingdom and the United States, though earlier on, China approved the CanSino vaccine for limited use in the military by June 2020 while Russia also approved the Sputnik V vaccine for emergency use in August 2020 and mass vaccination campaigns began world over shortly afterwards (7–9). With efforts of agencies like COVAX, an initiative formed by the WHO, GAVI, the Vaccine alliance and the coalition for epidemic preparedness innovations (CEPI), and the African Vaccine acquisition trust (AVAT), there has been a greatly improved availability of these vaccines in many developing nations including Nigeria (10, 11). In Nigeria, the first batch of vaccines arrived in March 2021 and we have further received over 10 million doses of COVID vaccine donation (12).

The very first targets for vaccination globally were healthcare workers (HCWs). These are those defined by the WHO as people whose job it is to protect and improve the health of their communities and are therefore also at most risk of contracting and disseminating the infection. In Nigeria, the National Primary Health Care Developmental agency (NPHCDA) commenced the first of the four projected phases of mass COVID-19 vaccination around the same time that the first batch of donated vaccines arrived. The first phase targeted HCWs, supporting staff, frontline workers and other first respondents (12).

COVID-19 vaccines received a mixed reception. While acceptance rate in some countries were very high: Ecuador 97%, China 85%, some other countries had very low acceptance rates: Jordan 28.4%, Kuwait 23.4% (13). The acceptance among HCWs only barely improved on that of the general populations in certain places and in fact was found to be less than that of the general population by some studies (14). This is a concerning situation as vaccination recommendation by HCWs has been demonstrated to significantly contribute to acceptance (15).

In Nigeria a couple of published works on willingness to accept the vaccine has put the acceptance rate at 66.2% (15), 55.5% (16), 48.6% (17), and 40% (18). Hesitancy rate of 50.5% was seen in a similar study on HCWs in Abia (19). The major predictors of hesitancy from these studies were younger age, female gender, single marital status, low level of education, low income level etc. Factors driving hesitancy include distrust in the government, spread of misinformation, fear of side effects and concern about vaccine efficacy.

In other climes, vaccine hesitancy rates among HCWs were similar to the Nigerian picture. A meta-analysis of willingness to accept COVID-19 vaccines carried out by Luo et al. among 24,952 HCWs revealed that the vaccination willingness was 51% (20). Another study carried out in among HCWs at the Istanbul University, Cerrahpasa Faculty of Medicine showed an acceptance rate of 66% (21). In Italy, a similar study involving 1155 HCWs showed that only 67% of the participants were willing to be vaccinated, citing lack of trust in vaccine safety as the major reason for refusal (22). Also another Egyptian study among HCWs in different regions showed an acceptance rate of 21%, while 28% flatly refused vaccination and 51% were undecided (23).

The purpose of this study is to assess the knowledge and acceptance of COVID-19 vaccines among HCWs in Enugu metropolis. This study will help estimate the acceptance rate of COVID-19 vaccines among HCW in Enugu state and identify factors responsible for this. As the COVID-19 pandemic continues to take new twists and turns with the emergence of new variants with different disease potentials, it may be important to furnish stake-holders with such wealth of evidence as this study aims to contribute so that they are able to strategize better in the fight to overcome the pandemic.

## Methods

### Study area and design

This study was a cross-sectional study conducted among HCWs in Enugu state, South-eastern Nigeria. The major tertiary hospitals located in Enugu metropolis are Enugu State University of Science and Technology Teaching Hospital Parklane, National Orthopaedic Hospital Enugu and Federal Neuropsychiatry hospital Enugu and were used for the study.

### Study population and procedure

HCWs in Enugu metropolis aged 18 years and above were engaged in the study. Questionnaires were distributed through the social media platforms (WhatsApp Channels) of the different hospitals in Enugu metropolis. Access to these channels were granted by the administrators of these platforms. Responses were obtained from individuals who voluntarily consented to participate by answering the questionnaires.

The minimum sample size was estimated to be 109, at a confidence interval of 95%. We had a total of 103 complete responses, which gives a response rate of 68.7%.

### Study duration

The study was conducted over a period of 3 months spanning from April to June, 2022.

### Data collection tool and methods

Data was collected using a self-administered semi-structured online-based questionnaire created on Google forms. The questionnaire design was guided by recommendations from the strategic advisory group of experts on immunizations (SAGE) vaccine hesitancy survey sample questions which were adapted to suit the Nigerian setting (24).

The questionnaires has 3 sections.

Section 1 assessed socio-demographic characteristics of the respondents including: age, sex, marital status, profession, educational status, and income. The subjective health status of the participants and their history of chronic illnesses were also established.

Section 2 assessed the knowledge of COVID-19 disease among respondents. It also requested for information on COVID-19 disease status of the respondents, their family members and their professional colleagues. This section also assessed for the respondent’s perceived risk of infection with COVID-19.

Section 3 assessed the respondents’ awareness of the availability of COVID-19 vaccines and nearby vaccination centres; acceptance of COVID-19 vaccines and reasons for refusal.

Measures taken in this study to limit research errors and bias associated with surveys include: randomization of the options, proper structuring and use of interval breaks was employed in in the questionnaires to limit answer order and agreement biases respectively; conduction of pilot studies was done prior to deployment of the survey tools to ensure suitability and accuracy of the questionnaire to the research objectives; we had a member of the research team follow up the responses and to respond to enquiries from the respondents to ensure accuracy of the answers and to improve response rate. We also had a panel created to oversee the data management and had two independent analysts work on the data to reduce systematic errors.

### Statistical analysis

Data analysis was carried out using Statistical package for Social Sciences (SPSS) by IBM version 22. The data was reviewed and cleaned before analysis. Descriptive analyses were conducted to determine frequencies and proportions of categorical variables in the total study sample. Then inferential analysis with the statistical significance set at *p* < 0.05 was employed after stratification by the yes/no answers to acceptance of COVID-19 vaccines. Chi-square tests were used to assess the association between different variables and acceptance of COVID-19 vaccines.

### Ethical considerations

Information obtained from the study was handled confidentially. Personal identification of respondents was precluded from the study tool. Respondents were informed that their participation was voluntary and consent was implied by completion of the questionnaire.

Ethics approval and consent to participate: ethical clearance was obtained from the University of Nigeria Teaching Hospital Health Research Ethics Committee with certificate number: UNTH/HREC/2022/06/462.

Consent for publication: participation was voluntary, and the purpose of the research was explained to each respondent. Informed consent was obtained before inclusion into the study. However, anonymity of participants was ensured, and no personal information was collected during the survey.

## Results

As shown in Table 1, the study received responses from a total of 103 HCWs. Majority of the respondents were females (55.3%). Most of the respondents were Christians (95.1%) and of the Catholic denomination. The majority of respondents were within 26–44 years age range (72.8%) and most were single (64.1%). All of the respondents had either completed the tertiary level of education (67%) or were at the postgraduate level (33%) and most respondents were either medical doctors (63%) or nurses (24%). Most also earned above #100,000 per month (67%) which is above the national minimum wage of #33,000.

**Table 1:** Socio-demographic characteristics of the study participants (*n* = 103).

Variable	Frequency *n* (%)
Age (y)	
18–25	26 (25.2)
26–44	76 (73.8)
45–60	1 (1.0)
>60	Nil
Sex	
Female	57 (55.3)
Male	46 (44.7)
Marital status	
Single	66 (64.1)
Married	36 (35.0)
Divorced	1 (1.0)
Widowed	Nil
Religion	
Christian	98 (95.1)
Muslim	4 (3.9)
Other	1 (1.0)
African traditional religion	Nil
If Christian, denomination	
Catholic	45 (45.9)
Pentecostal	33 (33.7)
Anglican	13 (13.3)
Methodist	2 (2.0)
Jehovah Witness	1 (1.0)
Presbyterian	1 (1.0)
Others	3 (3.1)
Level of education	
Tertiary	70 (68.0)
Post-graduate	33 (32.0)
Secondary	Nil
Primary	Nil
Occupation	
Medical doctor	65 (63.1)
Nurse	25 (24.3)
Medical laboratory scientist	4 (3.9)
Other	
Physiotherapist	9 (8.7)
Radiographer	Nil
Record staff	Nil
	Nil
Family income	
Above #100,000	69 (67.0)
Higher than average but less than #100,000	14 (13.6)
Lower than average	11 (10.7)
Average (#30,000)	9 (8.7)
Chronic illness	
Yes	14 (13.6)
No	89 (86.4)

Most of the respondents reported a very good subjective health status (86.4%). Majority of the respondents reported no history of chronic illness (86.4%) while among those with chronic illness (13.6%) the commonest were heart diseases and hypertension (7%), then respiratory diseases (5%).

**Table 2:** Reasons for vaccine refusal.

Reasons for refusing vaccine	*n*/*N* (*N* = Total)	Percentage
Fear of side-effects	30 (45)	66.7%
Concern about efficacy	29 (45)	64.4%
Lack of adequate information on available vaccines	18 (45)	40%
Preferred vaccine unavailable	5 (45)	11.1%
Religious reasons	3 (45)	6.7%
COVID-19 is not a dangerous disease	3 (45)	6.7%
Against vaccines in general	3 (45)	6.7%
Cultural reasons	1 (45)	2.2%

Most respondents believed COVID-19 to be a serious infection with the potential to cause death (82%). All were aware of the symptoms suggestive of COVID-19 infection.

About 42% reported a previous COVID-19 infection. While 57% either had not suffered or were not sure if they had suffered from a previous COVID-19 infections. More people however were aware of a family member or friend that had suffered from COVID-19 disease (59%).

The main sources of information on COVID-19 included a combination of social media, mass media, health conferences and seminars, interaction with families, friends and colleagues.

All the respondents confirmed that they had heard about COVID-19 vaccines. Nearly all were aware of nearby centres for COVID-19 vaccination (95%).

**Figure 1:**
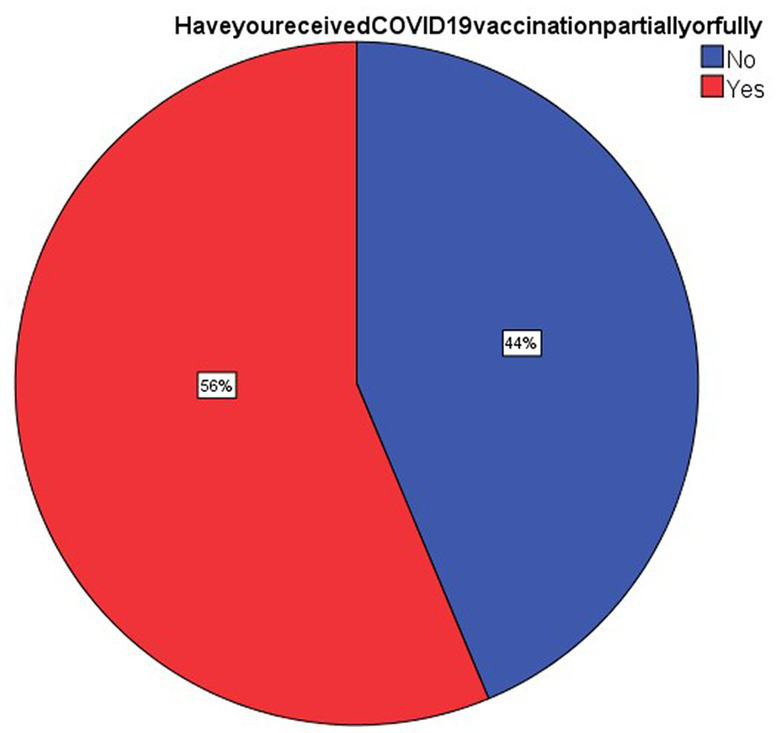
Data showing proportion of vaccine acceptance rate.

As can be seen in Figure 1 about 58 respondents (56.3%) had received the vaccines partially or fully. Hence the acceptance rate from this study is 56.3%. The predominant reason for receiving the vaccine was the belief that vaccination was protective against the infection (32%).

As noted in Table 3, the three major reasons for vaccine refusal/hesitancy were fear of side-effects (66.7%), concern about efficacy of the vaccines (64.4%) and lack of adequate information on the available vaccines (40%). Interestingly, few of the respondents (11%) who refused the vaccine indicated that they may be willing to vaccinate if the vaccines were paid for.

**Table 3:** Factors associated with acceptance of COVID-19 vaccine.

Factors	Not vaccinated (vaccination status)	Vaccinated	Chi-square	Value of p^*^
		(Vaccination status)		
Marital status				
Divorced	1	0		
Married	7	29		
Single	37	29	13.996	0.001
Age			13.161	0.004
18–25	19	7		
26–44	26	50		
45–59	0	1		
Income			10.766	0.013
Above #100,000	24	45		
Higher than average but below #100,000	10	4		
Average	3	6		
Lower than average	8	3		
Denomination			14.027	0.051
Anglican	11	2		
Catholic	19	26		
Jehovah Witness	1	0		
Methodist	1	1		
Pentecostals	11	6		
Presbyterian	0	22		
Others	2	1		
Religion	43	55	1.858	0.395
Christian	1	3		
Muslim	1	0		
Other				
Sex			1.532	0.216
Female	28	29		
Male	17	29		
Occupation			5.471	0.14
Medical doctor	23	42		
Medical laboratory Scientist	3	1		
Nurse	14	11		
Other	5	4		
Educational Level	10	23	4.568	0.102
Post graduate	35	35		
Tertiary				
Current State of health			0.419	0.518
Fairly good	5	9		
Very good	40	49		
Previous COVID-19 infection			4.677	0.096
No	25	21		
Not sure	6	7		
Yes	14	30		

*Value of *p* was derived using chi-square analysis on IBM SPSS version 21. Significant value of *p* is defined as values less than 0.05. The comparisons were based on vaccination status. **There were 103 respondents. 58 respondents have received the COVID-19 vaccines partially or fully while 45 respondents had not received the vaccines at all.

Table 3 shows factors associated with acceptance of COVID-19 vaccine. Age of the subjects was found to be significantly associated with acceptance of vaccine (*p* < 0.004) and Chi square value of 13.161. Age ranging 26 to 44 and above were more receptive of the vaccine compared to the younger age grouping 18–25 years.

More married people (80.6%) received the vaccines compared to singles (43.9%). There is a significant relationship between marital status and vaccination (*p* = 0.001, Chi square value = 13.996). Married individuals were more likely to receive the vaccines while single individuals were more likely not to receive the vaccines.

A 65% acceptance rate was noted among those with average monthly income above #100,000 compared to 29% among those with income higher than average but less than #100,000, 27% among those with income lower than average.

A significant correlation was found between average monthly income and vaccination (*p* = 0.013; Chi-square = 10.766).

Males (63.0%) showed greater acceptance of the vaccine than females (50.9%). The relationship between sex and acceptance of vaccine was however not significant (*p* = 0.216; Chi-square value: 1.532).

No significant association was found between educational level, religion, denomination nor occupation and acceptance of vaccine.

## Discussion

From our study, only slightly more than half of the HCWs accepted COVID-19 vaccination (56.3%). This finding is similar to that found in a study by Adejumo et al. (55.5%) (16) who also studied HCWs. It was also close to an acceptance rate of 51% determined by a meta-analysis on studies involving HCWs globally (20).

These acceptance rates fail to meet the minimum vaccination coverage of 75% per population predicted to establish herd immunity (25). Much more significant is the fact that this level of acceptance is found among the most medically literate sub-population in the country. This perhaps suggests that HCWs are affected by the same factors responsible for vaccine hesitancy in the general population.

Social demographic factors found to be positively associated with acceptance of vaccines and statistically significant included older age range, being married and having an average income above #100,000. This corresponds to findings among HCWs in Abia (19) and also with that of Uzochukwu et al. (26) in a Nigerian tertiary institution.

Acceptance rate was higher among males compared to females just like in the above studies. Sex was not found to be a statistically significant determinant of acceptance of the vaccine. Our finding corresponds to that of Adejumo et al. (16) In Nigeria, HCWs are among the most educated population hence we found that all the respondents had either a tertiary level of education or a post-graduate level.

As seen in Table 2, religious and cultural factors were found to not have any significant contribution to vaccine refusal. This finding is also similar to that of Adejumo et al. (16). While the medical training may not completely eliminate the factors that precipitate hesitancy to vaccines, it might have reduced the impact of religious and cultural influences on the decision to vaccinate or not.

A good number of respondents had either suffered from suspected COVID-19 disease or were aware of family, friends or colleagues that had suffered from the condition. More than two thirds of the respondents were concerned about getting infected by the virus. While concern/worry about infection was associated with increased acceptance of the vaccine, this relationship however was not statistically significant. This does not correspond to Adejumo et al. (16) who found the perceived risk of COVID-19 to be significantly associated with acceptance of vaccines. This may be because at the time of our study the morbidity patterns of COVID-19 have been better understood than earlier in the pandemic. Hence while concern about contracting the disease still remains, the fears may not be a strong enough motivation for vaccination compared to earlier during the pandemic.

Concern about side-effects, vaccine efficacy and lack of adequate information on the available vaccines were the leading reasons for refusal of vaccines. This finding is consistent with the findings of the study in Nnamdi Azikiwe University Awka (26) and other much more global studies on COVID-19 vaccine acceptance among HCWs (20, 27). This finding in this population considered most knowledgeable about healthcare conditions in general suggests that current information/education on COVID-19 disease and vaccines might still not be as convincing as needed. The level of misinformation and spread of conspiracy theories sustained a growing trajectory even as more information became available on the disease and about the vaccines (28). Perhaps this degree of misinformation and fallacious declarations seen during this pandemic affected the level of confidence in the vaccines. Other factors such as religious and cultural concerns, and a general hesitancy to all vaccines had no real impact on the acceptance of COVID-19 vaccine. This is in line with the finding by Adejumo et al. (16).

## Conclusion and recommendations

We have found the acceptance rate of COVID-19 vaccines to have fallen short of the minimum required to achieve herd immunity despite availability of vaccines and an awareness of nearby vaccination centres. This proportion among the health elite of the country portends a poorer outcome in the general population. The major reasons for refusal of vaccine all point to the prevailing atmosphere of COVID-19 misinformation and conspiracy theories. There is a need for stake-holders in the Nigerian public health sector to devise means to reasonably address present misconceptions and misinformation about the COVID-19 disease and vaccines. The approach necessarily needs to be adapted to become more open, targeted to specific groups based on their prevailing fears/concerns, engaging interactively with concerned individuals in order to disperse these fears with evidence-backed information.

## Data availability statement

The original contributions presented in the study are included in the article/supplementary material, further inquiries can be directed to the corresponding author.

## Ethics statement

The studies involving human participants were reviewed and approved by Health, Research and Ethical Committee of the University of Nigeria Teaching Hospital, UNTH, Enugu, Nigeria. The patients/participants provided their written informed consent to participate in this study.

## Author contributions

KI was the lead author, he conceived the idea of the research work, designed the questionnaire and analysis, was involved in the collation of the work, wrote the paper and did the editing and initial review of the manuscript. JA, CO, HU, OU, and OAO assisted with the collection of data and in the analysis of the work from the data. UA helped with reviewing the questionnaire and final manuscript. All authors contributed to the article and approved the submitted version.

## Conflict of interest

The authors declare that the research was conducted in the absence of any commercial or financial relationships that could be construed as a potential conflict of interest.

## Publisher’s note

All claims expressed in this article are solely those of the authors and do not necessarily represent those of their affiliated organizations, or those of the publisher, the editors and the reviewers. Any product that may be evaluated in this article, or claim that may be made by its manufacturer, is not guaranteed or endorsed by the publisher.
